# Population Dynamics of *Galerucella birmanica* and Its Aggregation Behavior in *Brasenia schreberi* Aquaculture System

**DOI:** 10.3390/insects16040371

**Published:** 2025-04-01

**Authors:** Yini Wang, Yahong Wang, Changfang Zhou

**Affiliations:** School of Life Sciences, Nanjing University, Nanjing 210023, China; wangyini0310@163.com (Y.W.); 502024300072@smail.nju.edu.cn (Y.W.)

**Keywords:** aquatic vegetable, *Brassica schreberi*, *Galerucella birmanica*, herbivore-induced plant volatiles, 2-phenylethyl isothiocyanate, plant–insect interaction

## Abstract

The aquatic vegetable *Brasenia schreberi* Gmel. (Nymphaeales: Cabombaceae), widely cultivated in China, faces severe challenges from pest disturbances. With a field investigation, *G. birmanica* occurred for several generations in the *Brasenia* mono-cultivation system. The first visual peak on leaf surfaces appeared in July, with eggs, larvae, pupae and adults all being recorded. The highest number of flying adults were trapped in August in reverse to the absence of beetles on leaves, followed by an extraordinary high number of eggs in September. *G. birmanica* exhibited a conspicuous aggregation in distribution, with severely chewed areas having a *G. birmanica* abundance 21.6 times that of the non-chewed areas. Laboratory studies with GC–MS and two-choice tests revealed four volatiles significantly discrepant in contents released from chewed/intact leaves: cis-3-hexenyl acetate (917.33 ± 29.56 vs. 604.034 ± 23.24 ng, chewed vs. intact), 2-phenylethyl isothiocyanate (595.37 ± 28.42 vs. 356.00 ± 13.44 ng), undecane (771.44 ± 34.72 vs. 1003.28 ± 47.88 ng) and methyl salicylate (1079.84 ± 49.39 vs. 532.11 ± 18.23 ng); among them, 2-phenylethyl isothiocyanate could significantly attract *G. birmanica* adults. Our study suggests that *G. birmanica* can be a severe threat to mono-cultivated *B. schreberi*, whereas the damaged leaves may sacrifice themselves by attracting the beetles with an herbivore-induced volatile, thus protecting the whole plant population from pest disturbance.

## Simple Summary

The aquatic macrophyte watershield, *Brasenia schreberi* Gmel., with its young buds coated in a thick mucilage served as a famous vegetable, has been cultivated in China for a long time. However, its production has been threatened by a pest, *Galerucella birmanica* Jacoby. This study investigated the population dynamics of *G. birmanica* throughout the entire growth season of *B. schreberi* from May to November and identified the aggregation behavior of the pest, with a preference for severely chewed leaf areas. Further analysis revealed that it was 2-phenylethyl isothiocyanate released from the damaged leaves that attracted *G. birmanica*. Our findings provide valuable insights for pest management in *B. schreberi* cultivation fields, and the attractant effect of 2-phenylethyl isothiocyanate on *G. birmanica* offers a new perspective for the development of attractants for this insect.

## 1. Introduction

The watershield, *Brasenia schreberi* Gmel., is a monotypic genus in the Cabombaceae family [1,2] (also listed in the Nymphaeaceae family in some references [3,4]). The perennial, floating-leaved aquatic macrophyte is widely but sporadically distributed in lakes and ponds across East Asia, Australia, West Indies, Africa and North and Central America, with the exception of Europe, where the species has been extinct [3,5]. The submerged shoots and leaves, especially young buds of the plants, are coated with thick mucilage containing multiple polysaccharides, like D-galactose, D-glucose, L-rhamnose, L-fucose, etc., and are considered for both nutritional and medical values [6,7,8]. Hence, young buds of *B. schreberi* have been served as a popular aquatic vegetable for a long time in China, Japan and some other eastern Asian countries, though only in China, *B. schreberi* are regularly farmed and harvested in shallow areas of ponds, lakes and irrigated fields with a water depth maintained at 30–80 cm [4,9].

Aquatic plants generally host a high diversity of insects on their leaf surfaces. Among which, the leaf beetle *Galerucella birmanica* Jacoby (Coleoptera: Chrysomelidae) is a holometabolous insect that has four stages, egg, larva, pupa and adult, with larvae and adults exhibiting a high consumption of leaves [10,11,12]. *G. birmanica* overwinters as an adult in weed residues and soil crevices on banks, starting activity in early April and moving to the overwintering sites in late October, with multiple and overlapping generations occurring annually in central and southern China [10,13]. *G. birmanica* prefers the water chestnut *Trapa natans* L. (Lythraceae) as its host [12,14], which grows in similar habitats as *B. schreberi* in natural aquatic areas. From late April to early May, when *T. natans* leaves emerge from the water, *G. birmanica* migrates to the leaves, feeds on the leaf tissue and enters a mating peak and lays eggs after 3–4 days [10]. Damaged *T. natans* leaves caused by *G. birmanica* exhibit numerous holes, and in severe cases, only the veins remain [13].

In North America, *G. birmanica* has been considered as a potential biocontrol agent against *T. natans*, which is a major invasive species in the continent [11,12,15]. Earlier research demonstrated that *G. birmanica* oviposited and was able to complete development on both *Trapa* spp. and *B. schreberi* in no-choice experiments [11], and in field choice tests, a strong preference for *T. natans* and only occasional “spill-over” onto *B. schreberi* were found. The recent report from Simmons and Blossey also suggested that the potential risk of *G. birmanica* towards *B. schreberi* was low [12]. However, with our field observation, we found that, in the mono-cultivation system of *B. schreberi* where the plants were harvested as a vegetable, the leaves suffered severe infestation by *G. birmanica*. Such infestation was unevenly distributed, with notable aggregation phenomena occurring in parts of the areas. Further research on the interaction between *G. birmanica* and *B. schreberi* is required, especially in mono-cultivation systems where *T. natans* is not available for the beetles.

The relationships between insects and their hosts are complicated. Plants have evolved multiple strategies to resist herbivory insects, including constitutive defense, induced response, etc. [16,17,18]. Reversely, insects may locate their hosts through visual, olfactory and gustatory cues, etc. [19,20,21,22]. For the olfactory cues, herbivorous insects may be attracted by specific components of plant volatiles. For example, nonanal emitted from tobacco during vegetative growth greatly attracted female *Helicoverpa assulta* (Lepidoptera: Noctuidae) [23]; (Z)-3-hexenyl-acetate emitted from maize attracted females of *Spodoptera frugiperda* (Lepidoptera: Noctuidae) and stimulated their oviposition [21]; cotton volatiles induced by the attack of *Anthonomus grandis* (Coleoptera: Curculionidae) were attractive to conspecifics [24]. Multiple volatile compounds from *B. schreberi* have been identified [25]; however, whether such volatiles function in the *Brasenia*–herbivore interaction remains unknown. In this study, we focused on the mono-cultivation system where *B. schreberi* was the only crop cultivated and no *T. natans* coexisted. We tried to verify if *G. birmanica* would accept *B. schreberi* as a substitute and to what extent this could occur. The monthly occurrence of the beetles with different developmental stages in the system through the whole growth period of *B. schreberi* was recorded. Additionally, by analyzing the volatile compounds emitted from *B. schreberi* leaves and their potential role as olfactory cues for the beetles, possible dynamics for the aggregation of *G. birmanica* in *B. schreberi* field were explored.

## 2. Materials and Methods

### 2.1. Study Location

The research field is located in Changshu Ecological Agricultural District of the Taihu Lake basin in East China (120°33′–121°03′ E, 31°33′–31°50′ N), which has the longest history of *B. schreberi* cultivation and remains one of the four major *B. schreberi* production areas of the country. A typical subtropical monsoon humid climate dominates in the area, with the annual average temperature being 15.4 °C, from an extreme minimum temperature of −11.3 °C to an extreme maximum temperature of 40.2 °C, and the annual average precipitation being 1135.6 mm. A human-maintained *B. schreberi* cultivation pond in the center of the district was selected for the survey of *G. birmanica*, where toxic pesticides have been forbidden for years before the study and only limited organic fertilizers are used. The pond is irrigated by the Wangyu River, which connects the third-largest freshwater lake, Taihu, and the longest river, Yangzte River, in China. The water depth in the pond is maintained at about 30 cm in early spring to help crop rejuvenation and about 60 cm in the other seasons for growth and overwintering. The total area of the pond is approximately 2400 m^2^.

### 2.2. Monitoring of Galerucella birmanica

**Figure 1:**
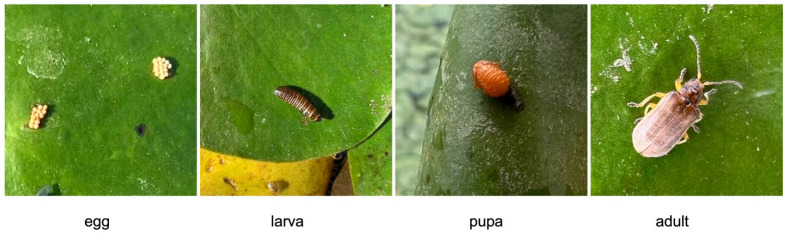
*Galerucella birmanica* at different developmental stages.

The presence and density of *G. birmanica* in the *B. schreberi* field were investigated by performing direct visual inspection of leaf surfaces and yellow board sticky trapping (25 cm × 20 cm, double-sided adhesive) once a month, covering the growth season of the crop from April to November 2023 (as no beetles were recorded in April, all data presented in this paper started in May). Three parallel transects with a distance no less than 10 m between one and another were set in the *B. schreberi* pond; along each transect, 12 sampling sites that were 4 m apart were marked, resulting in a total of 36 sites. At each site, the number of insects with different developmental stages within a 0.5 m × 0.5 m quadrat was first recorded by visual inspection on leaf surfaces. Since the eggs of the beetles were clustered together and the individual was too small to count, the number of eggs was recorded by every clutch (see the eggs in Figure 1; we treated them as 2 clutches). The number of larvae, pupae and adults was recorded by every individual, and the total number of beetles was counted as clutches of eggs plus individuals of the rests.

**Figure 2:**
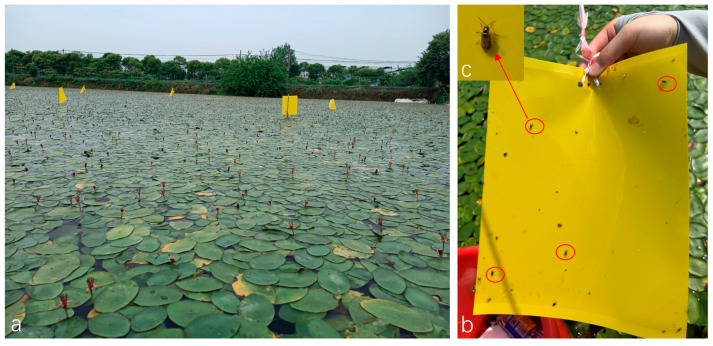
Sticky boards deployed in *Brasenia schreberi* field for trapping *Galerucella birmanica*. (**a**) yellow sticky boards deployed in the *B. schreberi* field; (**b**) one sample of the yellow sticky boards after deployed in the field for 24 h; (**c**) one sample of the adult *G. birmanica* captured on the sticky board.

As a supplement for the flying adults of *G. birmanica*, yellow sticky traps were temporarily placed at each sampling site immediately after visual inspection for an additional log of the number of *G. birmanica* (Figure 2). The trap was positioned as close to the water surface as possible, and after 24 h, the adults on the trap were counted.

Besides the beetles, the number of intact leaves and chewed leaves of *B. schreberi* was also counted, respectively, in each 0.5 m × 0.5 m quadrat; thus, the leaf chewed rate was calculated as

(1) $$leaf\;chewed\;rate=\frac{number\;of\;chewed\;leaves}{number\;of\;intact\;leaves+number\;of\;chewed\;leaves}\times 100\%$$

Then, seven leaves that exhibited the most severe chewed damage were selected to calculate the leaf damaged level:

(2) $$leaf\;damaged\;level=\frac{area\;of\;the\;damaged\;part\;of\;the\;leaf}{total\;area\;of\;the\;leaf}\times 100\%$$

The mean value of these calculations was used to represent the maximum extent of herbivore-induced damage at each sampling site.

### 2.3. Comparison of Galerucella birmanica Occurrence in Relatively Intact and Severely Chewed Areas

As visible aggregation of *G. birmanica* in the *B. schreberi* pond was noticed, two additional groups of quadrats, one in areas with relatively intact *B. schreberi* leaves and the other in areas where the leaves were severely chewed, were randomly marked in the pond in November of the same year. In each group, 42 quadrats were marked with an interval of at least 4 m, and then, the number of *G. birmanica* on the leaf surfaces was counted. As the total amount of beetles was reduced in November, each quadrat area here was enlarged to 2 m × 2 m.

### 2.4. Two-Choice Tests

To investigate the possible behavioral significance of adult *G. birmanica* in response to intact and chewed *B. schreberi* leaves, the volatiles from the two types of leaves were collected, followed by two-choice tests to evaluate beetle preferences.

For the collection of leaf volatiles, a headspace adsorption method was used [20,26]. Intact (non-chewed) and severely chewed leaves of *B. schreberi* were harvested from the field and taken back to the lab. They were kept moist and wrapped in a sampling bag made of heat-resistant nylon resin. The inlet of the sampling bag was connected to an air pump with a glass scrubber filled with 20/50 mesh activated-carbon adsorbent. The outlet of the sampling bag was connected to an adsorption column with 80/100 mesh Porapak Q (Supelco, Bellefonte, PA, USA). Teflon tubes were used for the accessory passages. The volatile collection for each sample was maintained for 24 h, with the gas flow rate at 400 mL min^−1^. Immediately after collection, the volatile sample on the column was eluted with chromatographic grade n-hexane (Aladdin Biochemical Technology Co., Ltd., Shanghai, China), and the eluent was then concentrated to 200–300 µL with nitrogen. A total of 200 ng of n-octane (Macklin Biochemical Co., Ltd., Shanghai, China) was added to each eluent as an internal standard. The collection for each type of leaf was repeated five times, with each sampling consisting of 15 leaves.

**Figure 3:**
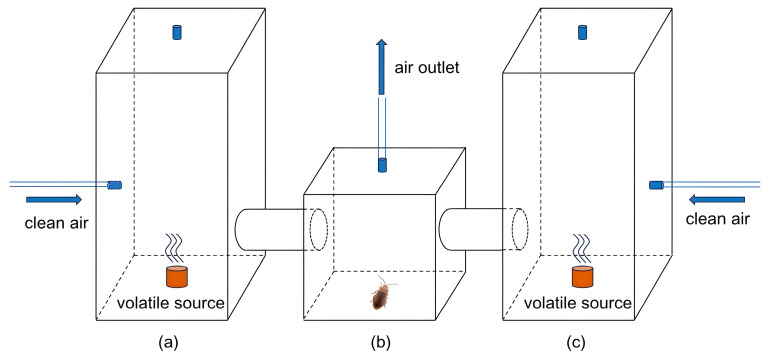
Three-chamber olfactometer. (**a**) left chamber with one of the volatiles; (**b**) middle chamber with the beetle; (**c**) right chamber with another volatile for compare.

The two-choice tests were based on the commonly used Y-tube olfactory test [27] but conducted in a self-designed, three-chamber olfactometer (Figure 3). First, one beetle was placed in the middle chamber (b) to adapt to the environment for 10 min. Then, 10 µL of the eluent from intact/chewed leaves of *B. schreberi* was applied to a 2 cm^2^ filter paper, which was placed in sequence into the left (a)/right (c) chamber of the olfactometer. Clean air at a flow rate of 500 mL min^−1^ was pumped into the chamber on each side of the olfactometer to make sure the beetle in the middle could perceive the odors from both sides. The air was pumped out from the top of the middle chamber (b) to ensure a controlled olfactory environment for the behavioral assays. The temperature in the olfactometer was maintained at about 25 °C during the tests. If the beetle remained in the selected chamber for more than 30 s, it was recorded as having made a choice for the volatile in that chamber. If the beetle remained in the initial middle chamber for 15 min without making a choice, it was considered to have no response and excluded.

The two-choice tests were repeated until 26 adult beetles were recorded to have made choices. To eliminate possible position effect, every 5 beetles that had made a choice were treated as one group; after each group, the positions of the filter papers with volatiles from intact/chewed leaves were swapped between the two sides of the olfactometer [27]. The system was ventilated for 30 min each time when swapping to remove possible residue. An additional blank assay was also carried out before the test to determine whether there was any difference in the beetle’s preference when both sides of the olfactometer contained only clean air.

### 2.5. GC–MS Analysis for Volatiles from Intact and Chewed B. schreberi Leaves

Gas chromatography–mass spectrometry (GC–MS, Agilent 6890 GC and 5975C MS, USA, equipped with an HP-5MS capillary column (30 m × 0.25 mm ID × 0.25 μm film thickness, Agilent, Santa Clara, CA, USA) was used to analyze the volatile components from intact and chewed *B. schreberi* leaves. Referring to the conditions described by Zhang et al. [20,26], the temperature was initially set at 40 °C for 5 min, increased to 185 °C at a rate of 5 °C min^−1^, further increased to 280 °C at a rate of 30 °C min^−1^ and then held for 1 min. Helium was used as the carrier gas at a flow rate of 1 mL min^−1^. The inlet temperature of GC–MS was set at 250 °C. The mass spectrometer was equipped with an electron ionization (EI) source, operating in full-scan mode across 30–500 m z^−1^. Temperature-controlled modules maintained the ion source at 230 °C and quadrupole at 150 °C, with acquisition parameters set to 3.2 scans s^−1^ and a 3 min solvent delay. We injected the samples using a 10 μL manual syringe, delivering 1 µL of concentrated *B. schreberi* volatile eluent through the front inlet. Volatile components were identified by comparison with the NIST 10.0 library. For quantification, we used the internal standard of octane, calculating the relative abundances from the total ion current (TIC) peak area ratios. All analyses were conducted using Agilent MassHunter Qualitative Analysis software 10.0.

### 2.6. Verification of Volatile Substance Attracting Galerucella birmanica

Four standard chemical solutions, cis-3-hexenyl acetate (Yuanye Bio-Technology Co., Ltd., Shanghai, China), 2-phenylethyl isothiocyanate (Bailingwei Chemical Technology Co., Ltd., Shanghai, China), undecane (Aladdin Biochemical Technology Co., Ltd., Shanghai, China) and methyl salicylate (Yuanye Bio-Technology Co., Ltd., Shanghai, China) in hexane of 1 µg µL^−1^, representing each of the volatiles that had exhibited remarkable discrepancy in contents between intact and chewed leaves, were prepared to further verify the choice preference of *G. birmanica*. Similarly, 10 µL of standard solution or hexane (as the control) on filter paper was placed in either side of the three-chamber olfactometer for selection by the beetle in the middle. Each standard solution was repeated until 26 beetles had made choices.

### 2.7. Statistical Analysis

Mann–Whitney U tests were used to compare the abundance of *G. birmanica* (both on leaves and on yellow sticky traps) between different months and between intact and severely chewed areas in November and were used for leaf damage conditions between different months. Spearman correlation analyses were performed on the degree of leaf damage and the abundance of *G. birmanica*. Paired sample *t*-tests were used to compare the differences in volatile contents between intact and chewed leaves. Chi-square tests were used to compare the two-choice test results of *G. birmanica* between volatiles of intact/chewed *B. schreberi* leaves and between reagents representing separated volatiles and the control.

## 3. Results

### 3.1. Monthly Occurrence of Galerucella birmanica in Brasenia schreberi Pond

**Figure 4:**
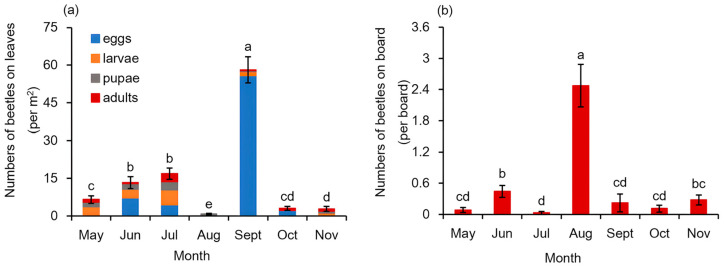
Abundance of *Galerucella birmanica* in different months. Bars represent standard error of immature and mature stages of beetles (**a**) or adult beetles (**b**), n = 36. Different letters represent a statistically significant difference (Mann–Whitney U test, *p* < 0.05).

With our field visual inspection, four typical developmental stages of *G. birmanica*, the egg, larva, pupa and adult, were found on the leaf surface of *B. schreberi* in the investigated pond (Figure 1). Their monthly occurrence on the leaf surfaces is shown in Figure 4a. *B. schreberi* in the research site started to rejuvenate in April, while no *G. birmanica* was found at the first month, when only sporadic leaves of the plants floated on the water surface. In May, when the leaves of the crop reached a coverage of more than 80%, the beetles on the leaves were first recorded, with relatively more larvae, followed by pupae and adults but no eggs. The total number of beetles on the leaves kept increasing until August, when the beetles almost disappeared during the hottest weather conditions. In September, the total beetle number on the leaves rebounded remarkably to 3.4 times that in July, with 96.0% of them being eggs. The beetles reduced quickly the following October and kept a limited occurrence in November, when the plants started to senesce. As for the different stages of the beetles, besides the remarkable peak of eggs in September, a small peak of eggs also appeared in June. Relatively abundant larvae, pupae and adults appeared in July.

As an additional record for the emergence of *G. birmanica* in the *B. schreberi* cultivation system, yellow sticky boards were deployed right after the visual inspection. The number of flying *G. birmanica* trapped on the boards showed a different trend compared to the direct record on the leaf surfaces. A significantly high number of adult beetles were recorded in August (Figure 4b). Small amounts of beetles were also trapped in June and November, but almost no beetles were trapped in May and July.

### 3.2. Leaf Damage Condition of Brasenia schreberi and Its Correlation with Galerucella birmanica Occurrence

**Figure 5:**
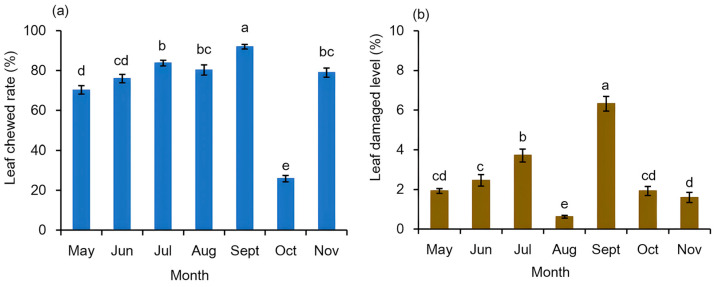
Leaf damaged condition of *Brasenia schreberi*. (**a**) Leaf chewed rate of *Brasenia schreberi*; (**b**) Leaf damaged level of *Brasenia schreberi*. Bars represent standard error, n = 36. Different letters represent a statistically significant difference (Mann–Whitney U test, *p* < 0.05).

Leaf damage condition was recorded at the same time of the beetles’ inspection. Since no pesticide was applied in the cultivation system, intact elliptic leaves of *B. schreberi* could only been found in April, when they first expanded on the water surface, and most of the leaves were more or less chewed in the subsequent seasons. Careful calculations revealed that 70.2% of the leaves were marked as being chewed in May (Figure 5a). The leaf chewed rate generally increased, peaking at 91.9% in September. October was an exception, as only 25.9% of the leaves were chewed. The chewed rate rebounded to over 70% in November.

Although most of the leaves showed signs of chewing damage, the average damaged level of each leaf was not so severe. As shown in Figure 5b, the leaf damaged level was lower than 2% in May, which increased to 3.7% in July and then decreased to the lowest point of 0.6% in August. The highest leaf damaged level occurred in September, as 6.3% of the leaf area was damaged. The leaf damaged level decreased to less than 2% in October and November.

**Figure 6:**
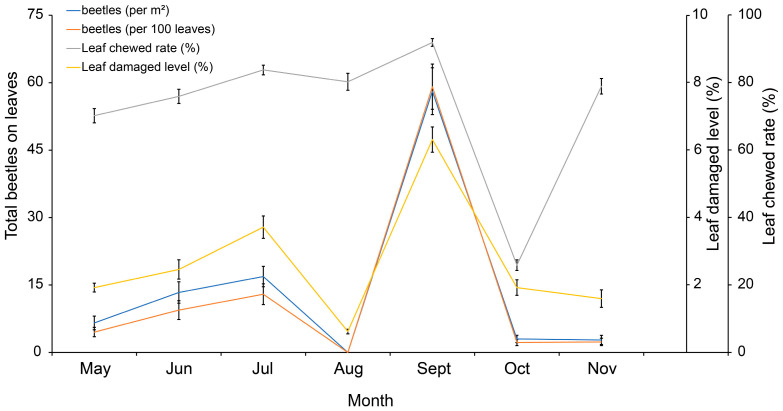
Correlation between leaf damage and beetle density. Bars represent standard error, n = 36.

The typical correlation between leaf damaged level of *B. schreberi* and *G. birmanica* occurrence on the leaf surfaces is shown in Figure 6. The trends of beetle numbers on the leaf surfaces through the growth season, both per m^2^ and per 100 leaves, closely mirrored the trend of leaf damaged levels. A visible but less remarkable correlation between leaf chewed rate and beetle numbers was also found.

With Spearman two-tailed tests, a detailed correlation between the occurrence of *G. birmanica* and the damage on *B. schreberi* leaves was further verified (Table 1). Positive correlations were found between the leaf chewed rate, leaf damaged level and number of beetles on the leaves, including eggs, larvae, pupae and total amount (*p* < 0.05); however, no significant correlation was found between the number of adult beetles on the leaves and leaf chewed rate (*p* > 0.05). The number of adult beetles trapped on the board was found to be positively correlated with the leaf chewed rate but negatively correlated with the leaf damaged level (*p* < 0.05). As for an additional calculation, the number of beetles per one hundred leaves on the leaf surfaces was significantly correlated with chewed damage (*p* < 0.05).

### 3.3. Aggregation of Galerucella birmanica in Brasenia schreberi Pond

**Figure 7:**
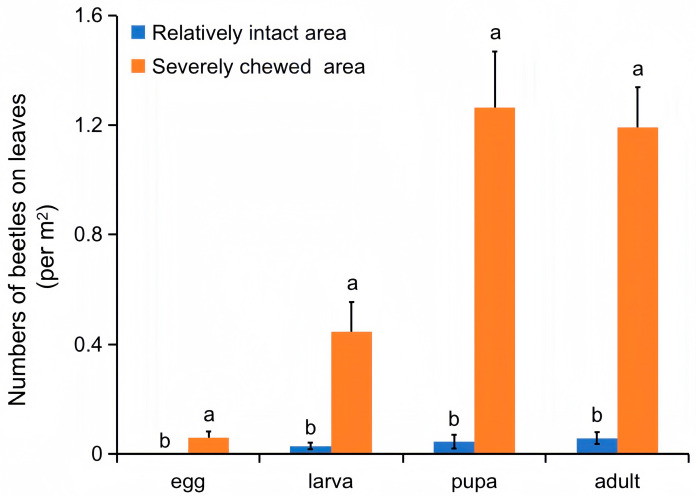
Beetles in the relatively intact and severely chewed areas. Bars represent standard error, n = 42. Different letters represent a statistically significant difference between intact and chewed areas (Mann–Whitney U test, *p* < 0.05).

The field investigation also found that the occurrence of *G. birmanica* in the *B. schreberi* pond was not uniform but inclined to aggregate in certain areas. Hence, an additional visual inspection was carried out in relatively intact/severely chewed areas in November. The number of *G. birmanica* at each insect stage was significantly higher in areas where *B. schreberi* leaves were severely chewed compared to that in areas where the leaves were relatively intact (*p* < 0.05), which not only further proved the correlation between the number of beetles and leaf damage but also indicated a certain mechanism for the aggregation of *G. birmanica* (Figure 7).

### 3.4. Volatiles Differed Between Intact and Chewed Brasenia schreberi Leaves

**Figure 8:**
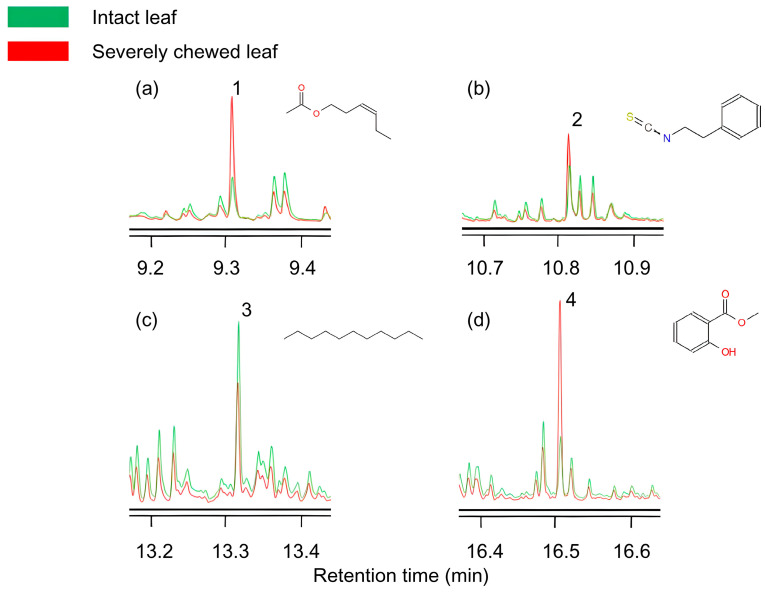
Four volatiles showing significant differences in contents between intact and severely chewed leaves analyzed by GC–MS. (**a**) 1, cis-3-Hexenyl acetate; (**b**) 2, 2-Phenylethyl isothiocyanate; (**c**) 3, Undecane; (**d**) 4, Methyl salicylate.

A GC–MS analysis was conducted to identify the components of the volatile samples collected from intact and severely chewed leaves of *B. schreberi*. Altogether, 51 volatile compounds as well as the internal standard octane were identified, among which, four components, cis-3-hexenyl acetate, 2-phenylethyl isothiocyanate, methyl salicylate and undecane, exhibited significant discrepancies in contents between intact and severely chewed leaves (Figure 8, Table 2). The emission of cis-3-hexenyl acetate, 2-phenylethyl isothiocyanate and methyl salicylate from severely chewed leaves was promoted by 51.9%, 67.2% and 102.9%, respectively, whereas the emission of undecane was reduced by 23.1% compared to those from intact leaves (*p* < 0.05).

### 3.5. Two-Choice Tests of Volatiles from Brasenia schreberi by Adult Galerucella birmanica

**Figure 9:**
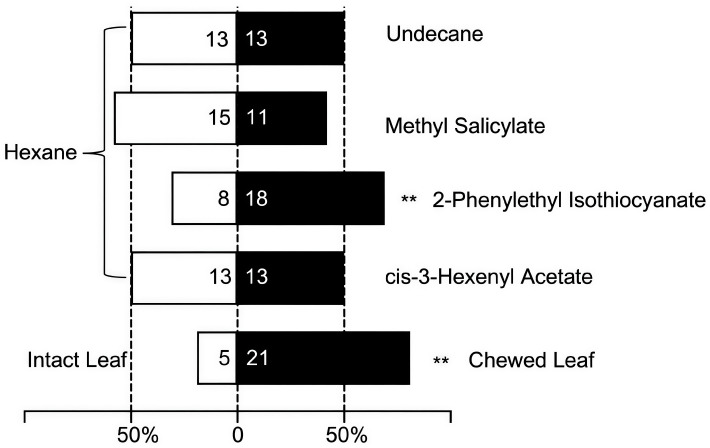
Two-choice tests of volatiles from *Brasenia schreberi* by *Galerucella birmanica* adults. ** represents *p* < 0.01 by chi-square tests, n = 26.

With the self-designed, three-chamber olfactometer, the volatiles from severely chewed and intact leaves of *B. schreberi* were extended to *G. birmanica* for choices. Strong preferences of the beetles were exhibited (Figure 9). Out of 26 adults, 21 of them selected the chamber with volatiles from severely chewed leaves, showing a statistically significant difference between the two types of leaves by the chi-square test (*p* < 0.01).

For the four components showing remarkable discrepancies in contents between volatiles from intact and severely chewed leaves, adult *G. birmanica* showed different performances (Figure 9). Using the solvent hexane as the control, a significantly higher number of the beetles tested showed a preference for 2-phenylethyl isothiocyanate (*p* < 0.01), whereas no preferences were found for cis-3-hexenyl acetate, methyl salicylate, undecane and the control (*p* > 0.05).

## 4. Discussions

Environmental factors strongly influence the seasonal variations in the population dynamics of insects. Temperature, in particular, plays a crucial role in the development and behavior of insects [28,29]. The detailed effect of temperature on the demography of *G. birmanica* has been studied in the laboratory [30]. In our field investigation, the population density of *G. birmanica* in the *B. schreberi* agricultural system exhibited significant seasonal variations. The population of *G. birmanica* generally increased from spring to summer, with temperature increasing, and reached its peak in September. The visual inspection data on leaf surfaces decreased dramatically in August, whereas the number of flying adults trapped by the sticky board was significantly high. Since the average temperature in our research site reached 29.2 °C in August and the daily highest temperature generally surpassed 30 °C, we suggest such high temperatures might stimulate the mating and oviposition of *G. birmanica* but inhibit egg development or impair egg viability, leading to the disappearance of *G. birmanica* of younger developmental stages on leaf surfaces in August but quantitative eggs in September. Similar patterns have been observed in other insects; for instance, when the temperature reaches 33 °C, the larval survival, pupation and adult emergence rates of *Diorhabda rybakowi* (Coleoptera: Chrysomelidae) all decrease significantly [31].

As for the discrepancy of *G. birmanica* peaks on leaf surfaces and sticky boards, we suppose such phenomenon was caused by the mating activity of the beetles. High temperature may stimulate the female or male *G. birmanica* to release sex pheromones. The beetles, in search of the pheromone source, would fly upwind along the pheromone gradient [32]. This also explains the significant number of eggs found in the next month.

The decrease in *G. birmanica* in October and November in the *B. schreberi* field might be caused by the lower temperature. Low temperature can inhibit the development of many insects [33]. For instance, the fecundity of *Mythimna separata* (Lepidotera: Noctuidae) and *Harmonia axyridis* (Coleoptera: Coccinellidae) was reduced, and their preoviposition period was extended under low temperatures [34,35]. Similarly, the feeding amount and fecundity of *Liriomyza huidobrensis* (Diptera: Agromyzidae) decreased significantly under low temperatures [36]. Afterwards, *G. birmanica* began its overwintering process.

Besides the temperature, rainfall might also affect the *G. birmanica* dynamics in the *B. schreberi* system. Our research site is characterized by a subtropical monsoon climate, and the *B. schreberi* field had just experienced a rainy season prior to our survey of the beetles in August. Heavy rain likely washed away many individuals previously present on the leaves. The lethal effect of rainfall on *G. birmanica* has been also reported by Lu et al. [10].

Based on the field observations of the *G. birmanica* population and leaf damaged condition of *B. schreberi*, we inferred that the first generation of adults likely originated from overwintering beetles near the pond, with the first batch of eggs laid in late April. Over the whole growth season of *B. schreberi*, there could be nine generations of *G. birmanica* that occurred in the system as follows: 1: late April–early June; 2: early June–early July; 3: early July–late July; 4: late July–early August; 5: early August–late August; 6: late August–early September; 7: early September–late September; 8: late September–mid-October; 9: mid-October–mid-November.

The trend of leaf damage of *B. schreberi*, both chewed rate and damaged level, corresponded to the fluctuations in the population of *G. birmanica*. Our findings confirmed that, in the absence of *T. natans*, *G. birmanica* treated *B. schreberi* as a substitute. Especially in areas like our research site, where *B. schreberi* is mono-cultivated as an important aquatic vegetable, *G. birmanica* poses a severe threat to the yield of *B. schreberi*. Therefore, careful consideration should be given before introducing *G. birmanica* as a biological agent to control the invasive *T. natans* in areas where *B. schreberi* is native.

For the uneven distribution of *G. birmanica* in the *B. schreberi* field, a similar aggregation scenario of *G. birmanica* has also been reported by Zheng et al. [37] with a *T. natans* system, and the authors suggested it may due to the biological characteristics of the beetles and the growth status of the plants. In our study, we revealed that damaged leaves of *B. schreberi* emitted increased amounts of cis-3-hexenyl acetate, 2-phenylethyl isothiocyanate and methyl salicylate but a decreased amount of undecane, among which, 2-phenylethyl isothiocyanate exhibited a strong attractant effect on *G. birmanica*. We suppose that the aggregation of *G. birmanica* is influenced by the plant volatile 2-phenylethyl isothiocyanate promoted by chewing. Isothiocyanates (ITCs) are typical herbivore-induced plant volatiles (HIPVs) that are produced when plant tissues are damaged by insect herbivory or mechanical injury [38].

In nature, the diverse array of HIPVs contributes to the complexity of plant defense and signaling processes [39]. ITCs generally play a crucial role in protecting plants from various pests and microorganisms [40], acting as deterrents and toxins [41], impairing the herbivore’s survival and growth and increasing their development time [42]. Whereas, in certain exceptional cases, ITCs emitted from plants can attract herbivores. Earlier reports suggested that the herbivore *Ceutorhynchus assimilis* (Coleoptera: Curculionidae) was attracted to ITCs in oilseed rape (*Brassica napus* L.) volatiles [43], and 3-butenyl, 4-pentenyl and 2-phenylethyl isothiocyanates might function as attractants to the pollen beetle *Meligethes aeneus* (Coleoptera: Chrysomelidae) [44]. In our research, 2-phenylethyl isothiocyanate, promoted in severely chewed *B. schreberi* leaves, acted as an attractant for the *G. birmanica*, indicating that *G. birmanica* has evolved to overcome the defense of *B. schreberi* through the utilization of ITCs. *G. birmanica* may track the 2-phenylethyl isothiocyanate as the signal of a food resource, mating partner or suitable habitat and thus aggregate in severely chewed areas in the *B. schreberi* pond. Similar attractant mechanisms of other HIPVs have also been found, as the leafminer *Tuta absoluta* (Lepidoptera: Gelechiidae) preferred infested tomato plants with up-regulated β-caryophyllene and tetradecane, supposed that infested plants might be more suitable for future larvae or might result in a decreased likelihood of predation or parasitism [45].

Cis-3-hexenyl acetate and methyl salicylate are also HIPVs that are frequently detected in plant–insect interactions [46,47]. Previous studies have demonstrated that both compounds were emitted by rice infested with *Nilaparvata lugens* (Hemiptera: Delphacidae), significantly enhancing parasitism rates of the parasitoid wasp *Anagrus nilaparvatae* (Hymenoptera: Mymaridae) on eggs of the planthopper [48]. The volatiles have been reported to trigger defense mechanisms both in the emitting plants and in neighboring plants [49,50]. Relatively higher levels of cis-3-hexenyl acetate and methyl salicylate were also detected in severely chewed *B. schreberi* leaves, whereas no preference was found for *G. birmanica* in two-choice tests. Such results may be caused by a specific gustatory perception of *G. birmanica*. Healthy leaves may also generate certain secondary metabolites that function as feeding deterrents to the beetles [46,51,52], meaning these compounds may cause the beetles to perceive a bitter taste or pungency, thereby discouraging them from feeding [47].

Undecane is a type of alkane plant volatile. A study with the common tobacco *Nicotiana tabacum* L. (Solanaceae) and its relative *Nicotiana benthamiana* Domin showed that a greater attraction of the latter to whitefly *Bemisia tabaci* (Hemiptera: Aleyrodidae) was due to a higher amount of the volatile undecane [53]. In another study, up-regulated undecane from *B. tabaci*-infested potato attracted more *Tuta absoluta* (Lepidoptera: Gelechiidae) [54]. With our study, the emission of undecane from severely chewed *B. schreberi* leaves was reduced. The mechanism for such down regulation was unknown, but still, undecane showed no preference for *G. birmanica*.

Altogether, 51 different volatiles from *B. schreberi* leaves were identified in our study, which were similar in amounts but differed substantially in composition compared to those reported by Zhang and Chen [25]. Such a discrepancy may be caused by different methods for sample collections. Leaf sources may also contribute to the discrepancy, as our sample was collected from East China, and their samples were from Central China. Differences could arise from plant genotypes, cultivation protocols (here, pesticides may be enrolled), environmental pollution, etc. Nevertheless, all four volatiles that showed significant differences in contents between chewed and intact leaves in our study have been nominated in other plant–insect interactions.

At the population or community level, herbivore damage to some leaves may promote defensive advantages to the entire plant and even their neighbors, with the latter being both conspecific and interspecific [55]. Our study indicates that, through the emission of 2-phenylethyl isothiocyanate, severely chewed *B. schreberi* leaves may sacrifice themselves to mitigate the harm of *G. birmanica* to other intact leaves. The neighboring defensive advantages are usually promoted by repelling signals that either resist the herbivore directly [56] or attract natural enemies [57], whereas in our study, 2-phenylethyl isothiocyanate acts as an attractant to the herbivore, further complicating the herbivore–plant interaction. Since *B. schreberi* is a clonal species and its rhizome in silt can extend for several meters, we still lack the evidence to verify if such defensive advantages only happen within leaves of the same individual or also extend to neighboring plants. As *G. birmanica* has been reported to prefer *T. natans* to *B. schreberi* when they coexist [11], it will also be interesting to explore if 2-phenylethyl isothiocyanate exhibits a further role in the interspecific neighboring effect between *B. schreberi* and *T. natans*.

From the point of view of *B. schreberi* aquaculture, artificially synthesized reagent 2-phenylethyl isothiocyanate may be considered as a potential attractant to control the burst of *G. birmanica*. HIPVs have been nominated in push–pull strategies to repel herbivores from crop plants while simultaneously attracting them to nearby trap plants [58]. Here, we suppose that the artificially synthesized reagent may play a similar role as natural HIPVs in aquaculture management. Compared with traditional pesticides that have been frequently used in *B. schreberi* aquaculture, like cypermethrin and permethrin, with notable toxicity that pollutes the food as well as the cultivation environment, 2-phenylethyl isothiocyanate, with its low dose and high attracting efficiency, can be a relatively safer substitute. Our research, with the population peaks of *G. birmanica* that burst along the growth period of *B. schreberi*, has indicated a proper time schedule for pest control measures. Further experiments are still expected for the detailed dose of 2-phenylethyl isothiocyanate that is needed in an open cultivation system.

## 5. Conclusions

In the mono-cultivation system of the aquatic vegetable *B. schreberi*, the leaf beetle *G. birmanica* caused severe damage to the floating leaves of the plants, with multiple generations occurring throughout the growth season of the crop and significant aggregation phenomena. Among the herbivore-induced plant volatiles released from damaged leaves of *B. schreberi*, 2-phenylethyl isothiocyanate could significantly attract adult *G. birmanica*, suggesting that the attacked plant leaves may sacrifice themselves to protect healthy neighboring leaves. The enhancement of the emission of 2-phenylethyl isothiocyanate is supposed to be a group defense strategy adopted by the crop to preserve population stability and to reduce the cost of individual plant defenses against *G. birmanica*.

## Figures and Tables

**Table 1:** Correlation between *Galerucella birmanica* abundance and leaf damage of *Brasenia schreberi*.

	Beetles on Leaves (per m^2^)					Beetles on Leaves (per One Hundred Leaves)	Beetles on Board
	Egg	Larva	Pupa	Adult	Total		
Leaf chewed rate	**0.3846** **(0.0000)**	**0.2416** **(0.0001)**	**0.2264** **(0.0003)**	0.1011 (0.1095)	**0.4533** **(0.0000)**	**0.4641** **(0.000)**	**0.1586** **(0.0117)**
Leaf damaged level	**0.6431** **(0.0000)**	**0.3919** **(0.0000)**	**0.3509** **(0.0000)**	**0.2691** **(0.0000)**	**0.7809** **(0.0000)**	**0.7783** **(0.0000)**	**−0.3034** **(0.0000)**

Note: Data shown here are R (*p*) values (boldface represents *p* < 0.05) analyzed by Spearman correlation analysis. Beetles on board only included adults, whereas those on leaves included all developmental stages.

**Table 2:** Volatiles emitted from *Brasenia schreberi* leaves (n = 5 pairs of severely chewed/intact leaves).

No.	Retention Time/Min	Compounds	CAS Number	Contents *^b^ (ng)		*t*-test *^c^ (*p* Value)
				Chewed Leaves	Intact Leaves	
1	3.959	Toluene	108-88-3	58.39 ± 4.14	58.97 ± 4.06	
2	4.499	Octane *^a^	111-65-9	200.00 ± 0.00	200.00 ± 0.00	
3	4.737	3-ethyl-Hexane	619-99-8	366.01 ± 24.49	365.64 ± 21.61	
4	5.988	Ethylbenzene	100-41-4	187.36 ± 26.85	179.84 ± 24.95	
5	6.185	p-Xylene	106-42-3	546.35 ± 55.36	543.52 ± 54.18	
6	6.714	Styrene	100-42-5	289.524 ± 23.44	289.64 ± 17.76	
7	7.046	Heptanal	111-71-7	230.112 ± 15.65	230.132 ± 12.64	
8	7.56	4-Ethylbenzoic acid	619-64-7	86.316 ± 12.50	101.932 ± 6.81	
9	8.559	Benzaldehyde	100-52-7	665.544 ± 24.83	670.554 ± 13.43	
10	8.722	1,2,3-trimethyl-Benzene	526-73-8	59.346 ± 3.94	58.646 ± 3.41	
11	8.893	(1S)-(1)-beta-Pinene	18172-67-3	161.38 ± 15.37	159.63 ± 16.62	
12	9.272	6-methyl-5-Hepten-2-one	110-93-0	276.52 ± 16.61	282.864 ± 10.41	
13	9.308	cis-3-Hexenyl Acetate	3681-71-8	917.33 ± 29.56	604.034 ± 23.24	0.001
14	9.552	Octanal	124-13-0	232.19 ± 22.16	223.388 ± 19.23	
15	9.822	Phenol	108-95-2	224.094 ± 17.12	226.398 ± 15.62	
16	10.289	D-Limonene	138-86-3	304.58 ± 21.10	300.4 ± 18.07	
17	10.357	2-ethyl-1-Hexanol	104-76-7	177.65 ± 9.31	176.19 ± 10.34	
18	10.826	2-Phenethyl isothiocyanate	2257-09-2	595.37 ± 28.42	356.00 ± 13.44	0.000
19	11.316	3-Carene	13466-78-9	189.00 ± 34.52	185.05 ± 30.89	
20	12.126	2,6,10-trimethyl-Dodecane	3891-98-3	222.22 ± 8.25	217.92 ± 8.77	
21	12.192	γ-Chlorobutyrophenone	939-52-6	287.82 ± 43.66	323.826 ± 22.37	
22	13.306	(E)-2-Hexenyl benzoate	76841-70-8	157.74 ± 11.90	142.974 ± 14.15	
23	13.337	Undecane	1120-21-4	771.44 ± 34.72	1003.28 ± 47.88	0.000
24	14.112	2-butyl-1-Octanol	3913-02-8	160.01 ± 14.42	158.91 ± 10.25	
25	14.261	Nonanal	124-19-6	175.60 ± 7.44	159.80 ± 8.25	
26	15.278	Camphor	76-22-2	154.43 ± 13.11	156.30 ± 11.13	
27	16.212	Naphthalene	91-20-3	184.13 ± 6.60	191.71 ± 7.12	
28	16.502	Methyl salicylate	119-36-8	1079.84 ± 49.39	532.11 ± 18.23	0.000
29	16.902	2,6,10-trimethyl-Tetradecane	14905-56-7	109.19 ± 22.45	113.38 ± 16.37	
30	17.234	Benzothiazole	95-16-9	171.96 ± 3.68	171.95 ± 2.95	
31	18.075	Stearic acid	57-11-4	142.30 ± 4.94	149.25 ± 4.72	
32	18.448	2-methyl-Undecane	97659-99-9	318.48 ± 26.72	314.55 ± 21.37	
33	18.421	Tridecane	629-50-5	377.56 ± 21.05	387.42 ± 11.08	
34	18.561	3-methyl-Tridecane	6418-41-3	107.24 ± 19.29	109.31 ± 17.34	
35	18.933	Oleic acid	112-80-1	372.65 ± 39.87	380.96 ± 31.36	
36	19.151	Tetradecane	629-59-4	200.07 ± 22.43	195.71 ± 11.36	
37	20.132	2-methyl-1-Hexadecanol	2490-48-4	579.88 ± 105.67	557.93 ± 35.53	
38	20.363	Dimethyl phthalate	131-11-3	211.96 ± 27.77	202.21 ± 17.90	
39	21.424	α-Farnesene	502-61-4	948.01 ± 8.00	947.66 ± 4.68	
40	21.569	Butylated Hydroxytoluene	128-37-0	1608.61 ± 164.07	1612.82 ± 131.27	
41	22.316	(Z)-9-Octadecenoic acid	8051-88-5	1766.76 ± 129.92	1743.13 ± 77.81	
42	22.903	Diethyltoluamide	134-62-3	101.82 ± 7.38	98.55 ± 5.20	
43	23.385	2-Dodecen-1-ylsuccinic anhydride	26544-38-7	256.41 ± 35.97	287.61 ± 29.21	
44	24.215	Behenic alcohol	661-19-8	512.80 ± 18.80	522.41 ± 11.97	
45	24.771	tert-Hexadecanethiol	25360-09-2	131.28 ± 12.63	137.69 ± 8.46	
46	25.134	Methyl cis-9,10-epoxyoctadecanoate	2566-91-8	336.41 ± 26.73	307.52 ± 13.27	
47	26.312	2,6-Ditert-butyl-4-ethylphenol	4130-42-1	157.00 ± 10.54	158.95 ± 12.13	
48	27.438	Albocycline	25129-91-3	112.61 ± 14.01	123.36 ± 6.15	
49	27.536	1-Heptatriacotanol	105794-58-9	150.99 ± 9.91	160.01 ± 11.11	
50	28.548	Obtusilactone	56799-51-0	186.20 ± 31.83	189.92 ± 10.50	
51	29.694	Dibutyl phthalate	84-74-2	725.29 ± 39.45	698.03 ± 20.26	
52	32.045	Heptacosane	593-49-7	472.62 ± 25.03	454.58 ± 21.81	

*^a^ Here, octane is the internal standard set at 200 ng in content; *^b^ relative contents of volatiles were calculated by comparing to that of octane; *^c^ for *t*-test results, only statistically significant differences were listed (*p* < 0.05) in the table, and blank means *p* > 0.05.

## Data Availability

The original contributions presented in this study are included in the article. Further inquiries can be directed to the corresponding author.
